# Dubious Claims about Simplicity and Likelihood: Comment on Pinna and Conti (2019)

**DOI:** 10.3390/brainsci10010050

**Published:** 2020-01-16

**Authors:** Peter A. van der Helm

**Affiliations:** Department of Brain & Cognition, University of Leuven (K.U. Leuven), Tiensestraat 102-Box 3711, B-3000 Leuven, Belgium; peter.vanderhelm@kuleuven.be

**Keywords:** contrast polarity, perceptual organization, simplicity principle, likelihood principle, simplicity–likelihood equivalence, Bayes, classical information theory, modern information theory

## Abstract

Pinna and Conti (*Brain Sci.*, 2019, *9*, 149, doi:10.3390/brainsci9060149) presented phenomena concerning the salience and role of contrast polarity in human visual perception, particularly in amodal completion. These phenomena are indeed illustrative thereof, but here, the focus is on their claims (1) that neither simplicity nor likelihood approaches can account for these phenomena; and (2) that simplicity and likelihood are equivalent. I argue that their first claim is based on incorrect assumptions, whereas their second claim is simply untrue.

## 1. Introduction

In the context of this journal’s special issue *Vividness*, *consciousness*, *and mental imagery: Making the missing links across disciplines and methods* [1], Pinna and Conti [2] presented fine phenomena concerning the salience and role of contrast polarity in human visual perception, particularly in amodal completion. They also claimed (1) that these phenomena go against existing simplicity and likelihood approaches to visual perception, and (2) that simplicity and likelihood are equivalent. Before they submitted their article to this journal, however, they had been informed that these claims are incorrect—a matter, by the way, of formal facts rather than psychological opinions. To set the stage, I first sketch the perceptual topic of their study.

## 2. Contrast Polarity

**Figure 1:**
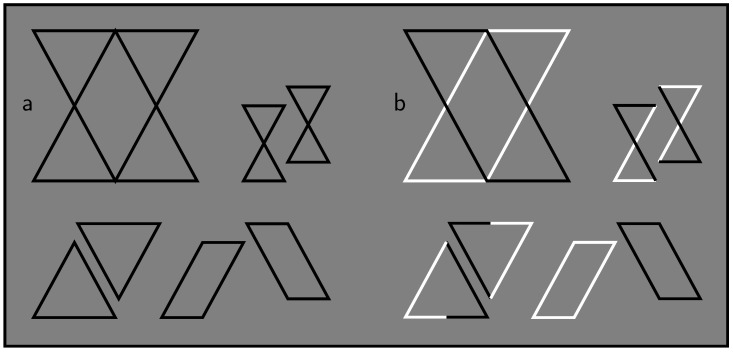
Contrast polarity and perceptual organization. Both compound stimuli can be described (i.e., interpreted) as consisting of, for instance, two triangles, two diabolos, or two parallelograms. The parallelogram interpretation is probably the weakest one in panel (**a**); but due to contrast polarity reversals, it is definitely the strongest one in panel (**b**) (after [8]).

The role of contrast polarity in human visual perception is a long-standing research topic (see, e.g., in [3,4,5,6,7]). As a consequence, the phenomena presented by Pinna and Conti are not really as novel or surprising as they suggested them to be, but they are indeed illustrative of the effects of, in particular, contrast polarity reversals. For example, Figure 1 shows, in the style of Pinna and Conti, a stimulus in which such a reversal triggers a substantial change in the way in which it is perceptually organized.

**Figure 2:**
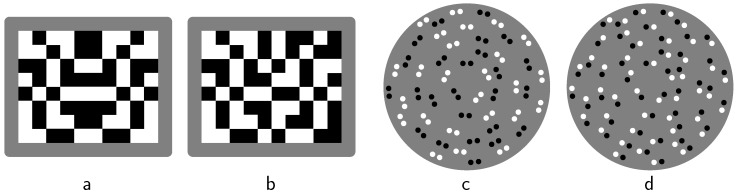
Contrast polarity and regularity detection. The symmetry in the checkerboard pattern in panel (**a**) is perceptually destroyed by the contrast polarity reversal in its right-hand half in panel (**b**); The Glass pattern in panel (**c**), with dipoles consisting of either two black or two white dots, exhibits a clear moiré effect, which disappears in panel (**d**), where all dipoles are identical with one black dot and one white dot.

Depending on stimulus type, contrast polarity also affects visual regularity detection. For instance, the evident symmetry in the checkerboard pattern in Figure 2a is perceptually destroyed by the contrast polarity reversal in its right-hand half in Figure 2b (see, e.g., in [9]). In dot patterns, however, such a reversal does not seem to do much harm (see, e.g., in [10,11,12]). Furthermore, Figure 2c depicts a rotational Glass pattern with dipoles consisting of either two black dots or two white dots. It exhibits a moiré effect that is perceptually about as strong as when all dipoles consist of black dots (personal observation). However, when all dipoles are identical with one black dot and one white dot, as depicted in Figure 2d, the moiré effect disappears (see, e.g., [4,13,14]). The moiré effect does not disappear, by the way, when every dipole consists of differently shaped elements [15].

Therefore, I agree with Pinna and Conti that contrast polarity is a factor to reckon with. However, I think that, in their first claim, they concluded too easily—in fact, based on incorrect assumptions—that contrast polarity triggers local groupings that precede the global groupings allegedly predicted by simplicity and likelihood approaches. Next, this is discussed in more detail.

## 3. Incorrect Assumptions

The following three quotes from Pinna and Conti illustrate their first claim, that is, their stance about contrast polarity versus simplicity and likelihood approaches:

“The salience and visibility, derived by the largest amplitude of luminance dissimilarity imparted by contrast polarity, precedes any holist or likelihood organization due to simplicity/Prägnanz and Bayes’ inference.”

[2] (p. 12 of 32)

“Contrast polarity was shown to operate locally, eliciting results that could be independent from any global scale and that could also be paradoxical. These results weaken and challenge theoretical approaches based on notions like oneness, unitariness, symmetry, regularity, simplicity, likelihood, priors, constraints, and past knowledge. Therefore, Helmholtz’s likelihood principle, simplicity/Prägnanz, and Bayes’ inference were clearly questioned since they are supposed to operate especially at a global and holistic level of vision.”

[2] (p. 26 of 32)

“The highlighting strength of contrast polarity determines even the grouping effectiveness against the global and holistic rules and factors expected by Helmholtz’s likelihood principle, simplicity/Prägnanz, and Bayes’ inference.”

[2] (p. 26 of 32)

It is true that simplicity and likelihood approaches may aim to arrive at global stimulus interpretations, but a general objection against the above stance is that they (can) do so by including local factors as well. For instance, van Lier [16] presented a theoretically sound and empirically adequate simplicity model for the integration of global and local aspects in amodal completion (see also [17]). A methodological objection is that Pinna and Conti introduced contrast polarity changes in stimuli but pitted these against alleged simplicity and likelihood predictions for the unchanged stimuli. As I specify next, this is unfair, and in my view, scientifically inappropriate.

### 3.1. Likelihood

Probabilistic approaches in cognitive science, and beyond, span a large spectrum (see Table 1, upper part, for a first impression thereof). At one end of this spectrum are Bayesian approaches like Friston’s free-energy predictive coding approach [18]. This approach claims to have high explanatory power, but in fact, hardly goes beyond data accommodation, hardly produces falsifiable predictions, and suffers from computational intractability (see, e.g., [19,20,21,22,23]). In my view, it therefore qualifies as what Chomsky called an “analogic guess”, that is, it “creates the illusion of a rigorous scientific theory with very broad scope” [24] (p. 32).

Most Bayesian models in cognitive science, however, take a stance that, in my view, is more appropriate and adequate. Instead of considering the likelihood principle as a strong explanatory principle, they rather consider it as a powerful modeling principle by which free-to-choose probabilities can be assigned to free-to-choose things in order to perform sophisticated data fitting and data extrapolation. This may not always be easy to do, but it means that there is no fundamental obstacle for the inclusion of local aspects like the effects of contrast polarity. Pinna and Conti wrote “[If] we do not consider the contrast polarity as a constraint or as a prior, Bayes’ inference cannot easily explain these conditions” [2] (p. 12 of 32)—indeed, but why would we? Therefore, they knowingly ignored the above flexibility and applied likelihood as if it is fundamentally blind to contrast polarity. Thereby, they missed the mark in their assessment of likelihood approaches.

### 3.2. Simplicity

Compared to the likelihood principle, the simplicity principle is less of a modeling principle and more of an explanatory principle. By this, I do not mean to claim that simplicity explains all contrast polarity phenomena. For instance, in Glass patterns, simplicity predicts stronger moiré effects for identical dipoles than for nonidentical ones, which may often be adequate but, as indicated, not in the case of Figure 2c,d. The point is that I consider simplicity to be a fundamental force in perception, which nevertheless—just as gravity in physics, for instance—interacts with other forces, yielding results that now and again may deviate from what simplicity on its own would yield.

In this respect, notice that the contrast polarity reversal in Figure 2b can be said to trigger local groupings which destroy the symmetry. It can also be said, however, to yield antisymmetry, which, on formal theoretical grounds within the simplicity paradigm, is predicted to be not one of the instantaneously detectable visual regularities [25,26]. The earlier mentioned reversal in dot patterns seems an exception to both rules [9]. Furthermore, the reversal in Figure 1 clearly implies that the parallelogram interpretation becomes less complex compared to the other two interpretations—provided one applies, unlike Pinna and Conti did, the simplicity idea correctly. This idea is specified next in some more detail (see Table 1, lower part, for a first impression thereof).

In both mathematics and perception research, the simplicity idea falls within a descriptive framework. It relies on regularity extraction to obtain simplest descriptive codes, which capture hierarchical organizations of given input. There are a few domain-dependent differences (see Table 1). Unlike algorithmic information theory in mathematics does, structural information theory in perception research employs a fixed descriptive coding language extracting theoretically and empirically grounded visual regularities, and it classifies things on the basis of the hierarchical organizations described by simplest descriptive codes (which are taken to reflect mental representations) [27,28,29,30,31,32,33,34,35].

A shared point, however, is that descriptive codes constitute reconstruction recipes for stimuli (just as computer algorithms are reconstruction recipes for the output they produce). Therefore, if a stimulus contains different contrast polarities, then these are necessarily also accounted for by descriptive codes of this stimulus. In Figure 1b, for instance, this implies that the contrast polarity changes in the triangles and diabolos make them more complex than the parallelograms without such changes are. Pinna and Conti knowingly ignored this and applied simplicity as if it is fundamentally blind to contrast polarity. Thereby, they missed the mark in their assessment of simplicity approaches.

### 3.3. Summary (1)

My objective here was not to show that simplicity and likelihood approaches can account for all contrast polarity phenomena (on their own, they certainly can account for some but probably not for all). Instead, my objective was to show that Pinna and Conti applied these approaches incorrectly, even though they had been warned about this. Thereby, they knowingly ignored that these approaches are far more flexible than they assumed them to be. In my view, this is scientifically inappropriate.

## 4. Simplicity and Likelihood Are Not Equivalent

Pinna and Conti formulated their second claim, about the alleged equivalence of simplicity and likelihood, as follows.

“[…] the visual object that minimizes the description length is the same one that maximizes the likelihood. In other terms, the most likely hypothesis about the perceptual organization is also the outcome with the shortest description of the stimulus pattern.”

[2] (p. 3 of 32)

This is an extraordinary claim. It therefore requires extraordinary evidence, but Pinna and Conti actually provided no corroboration at all (in their earlier draft, they cited Chater [46]; see Section 4.1. Instead, they seem to have jumped on the bandwagon of an idea that, for the past 25 years, has lingered on in the literature—in spite of refutations. As said, Pinna and Conti had been informed about its falsehood but chose to persist. It is therefore expedient to revisit the alleged equivalence of simplicity and likelihood (see Table 1 for a synopsis of relevant issues and terminologies).

Before going into specific equivalence claims, I must say that, to me, it is hard to even imagine that simplicity and likelihood might be equivalent. Notice that descriptive simplicity is a fairly stable concept. That is, as has been proved in modern information theory (IT) in mathematics, every reasonable descriptive coding language yields about the same complexity ranking for things [43,44,45]. Probabilities, conversely, come in many shapes and forms. For instance, on the one hand, in technical contexts like communication theory, the to-be-employed probabilities may be (approximately) known—though notice that they may vary with the situation at hand. For known probabilities, one may aim at minimal long-term average code length for large sets of identical and nonidentical messages (i.e., Shannon’s [38] optimal coding), and by the same token, at compounds of label codes that yield data compression for large compounds of identical and nonidentical messages (see, e.g., in [47,48]). On the other hand, the Helmholtzian likelihood principle in perception is now and again taken to rely on objective “real” probabilities of things in the world. This would give it an explanatory nature, but by all accounts, it seems impossible to assess such probabilities (see, e.g., in [49,50]). In between are, for instance, Bayesian models in cognitive science. In general, as said, such models employ free-to-choose probabilities for free-to-choose things, where both those things and their probabilities may be chosen subjectively on the basis of experimental data or modeller’s intuition. Therefore, all in all, how could one ever claim that fairly stable descriptive complexities are equivalent to every set of probabilities employed or proposed within the probabilistic framework?

Yet, notice that Pinna and Conti are not alone in their equivalence claim. Equivalence also has been claimed by, for instance, Friston [51], Feldman [52,53], and Thornton [54]. They too failed to provide explicit corroboration, which raises the question of where the claim actually comes from. As a matter of fact, for alleged support, they all referred consistently to either Chater [46] or MacKay [55], or to both. These sources are discussed next (for more details, see in [17,22,56,57]).

### 4.1. Chater (1996)

**Figure 3:**
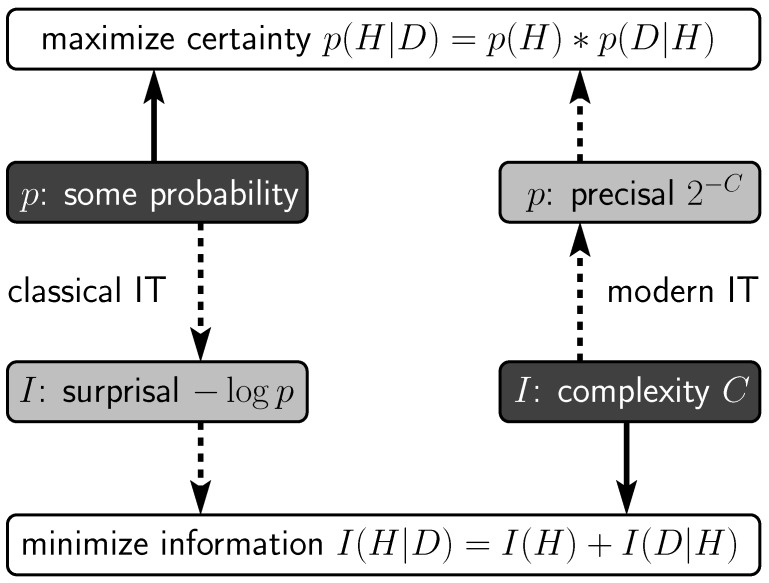
Surprisals versus precisals. For data *D* and hypotheses *H*, probabilities *p* can be used to maximize Bayesian certainty under these probabilities (top left), and via the surprisal conversion I=−logp, also to minimize information as quantified in classical information theory (IT) (bottom left). Descriptive complexities *C* can be used to minimize information as quantified in modern IT (bottom right), and via the precisal conversion $p=2^{-C}$, also to maximize Bayesian certainty under these precisals (top right) (adapted from [57]).

The main issue in the well-cited article by Chater [46] may be explained by means of Figure 3, starting at the left-hand side. The upper-left quadrant indicates that, for some set of probabilities *p*, one can maximize certainty via Bayes’ rule, that is, by combining prior probabilities p(H) and conditional probabilities p(D|H) for data *D* and hypotheses *H* to obtain posterior probabilities p(H|D). [*Note:* in general, priors account for viewpoint-independent aspects (i.e., how good is hypothesis *H* in itself?), whereas conditionals account for viewpoint-dependent aspects (i.e., how well do data *D* fit hypothesis *H*?).] The lower-left quadrant indicates information measurement in the style of classical IT, that is, by the conversion of probabilities *p* to surprisals I=−logp (term coined by Tribus [58]; concept developed by Nyquist [36] and Hartley [37]). As said, the surprisal can be used to achieve optimal coding [38], but as indicated in Figure 3, prior and conditional surprisals can, analogous to Bayes’ rule, also be combined to minimize information as quantified in classical IT. The latter constitutes the minimal message length principle (MML) [40], which, considering the foregoing, clearly is a full Bayesian approach that merely has been rewritten in terms of surprisals [59].

Turning to the right-hand side of Figure 3, the lower-right quadrant indicates that, for some descriptive coding language yielding complexities *C*, one can combine prior and conditional complexities to minimize information as quantified in modern IT. This is the minimum description length principle (MDL) [42], which can be seen as a modern version of Occam’s razor [60]. It also reflects the current take on the simplicity principle in perception [16,17]. The upper-right quadrant indicates that complexities *C* can be converted to what are called algorithmic probabilities $p=2^{-C}$, also called precisals [17]. These are artificial probabilities but, just as holds for other probabilities, prior and conditional precisals can, for instance, be combined to maximize certainty via Bayes’ rule. This reflects Solomonoff’s [44,45] Leitmotif: because classical IT relies on known probabilities, he wondered if one could devise “universal” probabilities, that is, probabilities that can be used fairly reliably whenever the actual probabilities are unavailable. In modern IT, precisals are proposed to be such universal probabilities and much research goes into their potential reliability. In cognitive science, they can be used, for instance, to predict the likelihood of empirical outcomes according to simplicity (i.e., rather than assuming that the brain itself uses them to arrive at those outcomes).

The surprisal and precisal conversions are convenient in that they allow for sophisticated theoretical comparisons between simplicity and likelihood approaches (see, e.g., in [59,60]). Chater, however, jumped to the conclusion that these conversions imply that simplicity and likelihood are equivalent. Notice that the left-hand and right-hand sides in Figure 3 represent fundamentally different starting points and lines of reasoning. Therefore, equivalence would hold only if, in the lower half, the left-hand probability-based quantification of information and the right-hand content-based quantification of information—or, in the upper half, the related left-hand and right-hand sets of probabilities—are identical. Apart from the fundamental questionability thereof, these were not issues Chater addressed. It is true that the conversions imply that simplicity and likelihood can use the same minimization and maximization formulas, but Chater fatally overlooked that equivalence depends crucially on what they substitute in those formulas—here, it is clear that they substitute fundamentally different things. Chater’s mistake is in fact like claiming that Newton’s formula ma for force *F* is equivalent to Einstein’s formula $mc^{2}$ for energy *E*—allegedly because both could have used a formula like mX, but fatally ignoring that *X* is something fundamentally different in each case. Therefore, all in all, Chater provided no evidence for equivalence of simplicity and likelihood at all.

### 4.2. MacKay (2003)

In what soon became a standard Bayesian textbook, MacKay [55] devoted one chapter (Chapter 28) to links between simplicity and likelihood. He actually did not claim equivalence, but as I discussed in [57] and revisit here, he mistakenly equated surprisals and description lengths, and he made an admittedly compelling argument that, however, was overinterpreted by others—who, subsequently, did claim equivalence.

One of MacKay’s conclusions was that “MDL has no apparent advantages over the direct probabilistic approach” [55] (p. 352). However, he attributed MDL not to MDL developer Rissanen [42] but to MML developers Wallace and Boulton [40]—just as [61] later did too, by the way. In fact, in the entire chapter, Mackay mistakenly wrote “MDL” instead of “MML” and “description length” instead of “message length” or “surprisal” (Baxter & Oliver [62] noticed this mistake also in MacKay [63]). Therefore, he in fact discussed the Bayesian MML and not the non-Bayesian MDL. No wonder, therefore, that he saw “no apparent advantages”. Unfortunately, his mistake added to the already existing misconceptions surrounding simplicity and likelihood. For instance, subsequently, Feldman [53,64,65,66,67] also mixed up MDL’s description lengths (which, i.t.o. modern IT’s descriptive codes, aim at minimal code length for individual things) and MML’s surprisals (which, i.t.o. classical IT’s label codes, minimize long-term average code length for large sets of identical, and nonidentical things).

MacKay’s mistake above already may have triggered equivalence claims, but unintentionally, another conclusion may have done so more strongly. That is, he also argued that “coherent inference (as embodied by Bayesian probability) automatically embodies Occam’s razor” [55] (p. 344). This is easily read as suggesting equivalence (see, e.g., in [52,53]), but notice that MacKay reasoned as follows.

“Simple models tend to make precise predictions. Complex models, by their nature, are capable of making a greater variety of predictions […]. So if $H_{2}$ is a more complex model [than $H_{1}$], it must spread its predictive probability $P(D|H_{2})$ more thinly over the data space than $H_{1}$. Thus, in the case where the data are compatible with both theories, the simpler $H_{1}$ will turn out more probable than $H_{2}$, without our having to express any subjective dislike for complex models.”

[55] (p. 344)

In other words, he argued that conditional probabilities, as used in Bayesian modeling, show a bias towards hypotheses with low prior complexity. This is definitely interesting and compelling, and as he noted, it reveals subtle intricacies in Bayesian inference.

Currently relevant, however, is that it does not imply equivalence of simplicity and likelihood. For instance, regarding both priors and conditionals, it is silent about how close (fairly stable) simplicity-based precisals and (fairly flexible) Bayesian probabilities might be. Furthermore, whereas prior precisals are nonuniform by nature, MacKay explicitly assumed uniform prior probabilities (he needs this not-truly-Bayesian assumption, because nonuniform prior probabilities could easily overrule the bias he attributed to conditional probabilities). This assumption as such already excludes equivalence. Notice further that he neither gave a formal definition of complexity nor a formal proof of his argument. This means that his argument, though certainly compelling, does not reflect a formally proven fact. Thereby, it has the same status as, for instance, van der Helm’s [17] argument that, specifically in visual perceptual organization, simplicity-based conditional precisals are close to intuitively real conditional probabilities—which would imply that precisals are fairly reliable in the everyday perception by moving observers. It is true that both arguments reflect interesting rapprochements between simplicity and likelihood, but neither argument asserts equivalence.

### 4.3. Summary (2)

My objective here was to trace back where Pinna and Conti’s misguided equivalence claim came from. This led to Chater [46] and MacKay [55], whose flawed comprehension of the links between classical IT and modern IT seems to have given rise to various misconceptions. It is true that they pointed at interesting things, but they did not provide any evidence of equivalence of simplicity and likelihood. With fundamentally different baits, classical IT and modern IT are fishing in the same pond of probabilities and information measurements—using a perhaps mind-boggling body of terms. It is therefore understandable that comparisons between them may be confusing, particularly to those who are less trained in formal reasonings. Persisting in an equivalence claim after having been informed in detail that such a claim is nonsense—as Pinna and Conti did—is another matter however, and in my view, scientifically inappropriate.

## 5. Conclusions

In this comment, I revisited the claims put forward by Pinna and Conti. First, they argued that simplicity and likelihood approaches cannot account for the contrast polarity phenomena they presented. I showed, however, that their argument was based on incorrect assumptions and that simplicity and likelihood approaches are far more flexible than they assumed them to be—without claiming, by the way, that they can account for all contrast polarity phenomena. Second, even though it did not seem essential in their article, they argued that simplicity and likelihood are equivalent. I showed, however, that, although this issue is prone to confusion, there is no reason whatsoever to suppose that simplicity and likelihood might be equivalent. Considering that this is a matter of formal facts rather than psychological opinions, it is, in my view, worrying that—in spite of refutations—unsubstantiated equivalence claims linger on in the literature.

## Figures and Tables

**Table 1:** Overview of probabilistic and descriptive frameworks.

*Probabilistic framework*
Classical information theory:
• information load of thing with probability *p* is surprisal −log(p) [36,37] (information quantified by its probability, not by its content)
• codes are nominalistic labels referring to things (as, e.g., in the Morse Code)
• optimal coding by label codes the length of surprisals [38] (implying minimal long-term average code length, not individually shortest codes)
Likelihood principle [39]:
• preference for things with higher probabilities, i.e., with lower surprisals
Bayesian inference (*incomputable* [21]):
• in cognitive science: free-to-choose probabilities for free-to-choose things
• minimum message length principle (message length measured i.t.o. surprisals) [40]
Surprisals do not enable descriptive formulation
*Descriptive framework*
Modern information theory (triggered by the question: what if probabilities are unknown?):
• codes are hierarchical descriptions (i.e., reconstruction recipes) of individual things
• shorter descriptive codes by extracting regularities i.t.o. identity relationships between parts
• information load of thing is its complexity *C*, i.e., the length of its shortest descriptive code (information quantified by its content, not by its probability)
Simplicity principle:
• a.k.a. minimum principle [41] or minimum description length principle [42]
• preference for simpler things, i.e., things with shorter descriptive codes
Algorithmic information theory (*mathematics*) [43,44,45]:
• extraction of any imaginable regularity (*incomputable*)
• classification by complexity of simplest descriptive code
Structural information theory (*cognitive science*) [27,28,29]:
• extraction of theoretically and empirically grounded visual regularities (*computable*) [30,31,32,33]
• classification by hierarchical organization described by simplest descriptive code [34,35]
Precisals $2^{-C}$, a.k.a. algorithmic probabilities, enable probabilistic formulation
